# The Back Muscle Surface Electromyography-Based Fatigue Index: A Digital Biomarker of Human Neuromuscular Aging?

**DOI:** 10.3390/bioengineering10030300

**Published:** 2023-02-27

**Authors:** Gerold Ebenbichler, Richard Habenicht, Peter Blohm, Paolo Bonato, Josef Kollmitzer, Patrick Mair, Thomas Kienbacher

**Affiliations:** 1 Karl-Landsteiner-Institute of Outpatient Rehabilitation Research, 1230 Vienna, Austria; 2 Department of Physical Medicine, Rehabilitation and Occupational Medicine, Medical University of Vienna, General Hospital of Vienna, 1090 Vienna, Austria; 3 Department of Physical Medicine and Rehabilitation, Harvard Medical School, Spaulding Rehabilitation Hospital, Boston, MA 02129, USA; 4 Department of Biomedical Engineering, TGM College for Higher Vocational Education, 1200 Vienna, Austria; 5 Department of Psychology, Harvard University, Cambridge, MA 02138, USA

**Keywords:** aging, low back pain, surface electromyography, muscle fatigue, cyclic exercise

## Abstract

As part of our quest for digital biomarkers of neuromuscular aging, and encouraged by recent findings in healthy volunteers, this study investigated if the instantaneous median frequency (IMDF) derived from back muscle surface electromyographic (SEMG) data monitored during cyclic back extensions could reliably differentiate between younger and older individuals with cLBP. A total of 243 persons with cLBP participated in three experimental sessions: at baseline, one to two days after the first session, and then again approximately six weeks later. During each session, the study participants performed a series of three isometric maximal voluntary contractions (MVC) of back extensors using a dynamometer. These were followed by an isometric back extension at 80% MVC, and—after a break—25 slow cyclic back extensions at 50% MVC. SEMG data were recorded bilaterally at L5 (multifidus), L2 (longissimus dorsi), and L1 (iliocostalis lumborum). Linear mixed-effects models found the IMDF-SEMG time-course changes more rapidly in younger than in older individuals, and more prominently in male participants. The absolute and relative reliabilities of the SEMG time–frequency representations were well compared between older and younger participants. The results indicated an overall good relative reliability, but variable absolute reliability levels. IMDF-SEMG estimates derived from cyclic back extensions proved to be successful in reliably detecting differences in back muscle function in younger vs. older persons with cLBP. These findings encourage further research, with a focus on assessing whether an IMDF-SEMG-based index could be utilized as a tool to achieve the preclinical detection of back muscle aging, and possibly predict the development of back muscle sarcopenia.

## 1. Introduction

The decline in back muscle strength and power (i.e., the rate of muscle force generation) with age may translate into disablement and adverse health outcomes [1]. Aging may affect the neuromuscular system in a complex fashion, often associated with changes in muscle structure, and a decline in muscle function (i.e., muscle strength, power, and endurance) [1,2]. MRI imaging studies suggest that neuromuscular aging processes particularly affect the back extensor muscles, and they start already at the end of the fourth decade [3,4,5]. As back extensors are critically involved in gait and balance function, the early functional decline associated with aging may result in an elevated risk of falls, disability, and dependency [6,7,8,9,10]. Low physical activity level is a key manifestation of the aging-related loss of muscle function [1,2], frequently present in persons with chronic low back pain (cLBP). Non-specific cLBP refers to pain in the lower back without a proven cause, and includes patients who present with imaging-based degenerative features of the spinal column [11,12]. As cLBP is the most prevalent health condition with epidemic-like dimensions in all age groups, it would be desirable to identify the digital biomarkers of aging-related decline in (neuro)muscular function, particularly from the back extensor muscles in this specific population. Detecting early signs of neuromuscular decline with non-invasive, sensitive, and predictive digital biomarkers could provide opportunities for intervention to prevent or to delay both external and internal risk factors affecting progression, and thus disability, dependency, and early death [1,13,14,15,16,17].

We recently suggested that an index derived from surface electromyographic (SEMG) data collected during a fatigue test could be used as a digital biomarker of the elevated risk of an early onset of neuromuscular aging processes [18,19]. This method estimates—via a time–frequency transformations—the compression over time of the SEMG frequency spectrum associated with localized muscle fatigue. Fatigue-related shifts of the spectral components of the SEMG data recorded from high load sustained contractions where the pool of recruitable motor units is activated and muscle blood flow is occluded are suggested to be predominantly mediated by hydrogen ion accumulation resulting from anaerobic metabolic pathways [20,21]. Consequently, the change in spectral components of the SEMG during such sustained high load static or slow isotonic cyclic contractions would predominantly relate to both the proportion and the metabolic capacity of glycolytic muscle fibers activated, as well as the firing rate relative to the fusion rate, within the detection volume of the SEMG sensors [22,23,24,25]. Given that potential confounders are controlled for (e.g., the relative displacement of the surface electrodes and active muscle fibers, which is accounted for when using time–frequency-based techniques), an age-related decline in glycolytic muscle metabolic capacity, which may relate to a loss of type II muscle fibers in aging muscles, would likely account for the shift toward the lower frequency of the SEMG frequency content [22,26].

Test protocols relying on the collection of data during isometric 80% MVC back extensions sustained for approximately 30 s have traditionally been used to derive the MDF-SEMG time-course [23,27]. However, as these measures represent a close to maximal task, they bear an elevated risk of back injury and medical complications in persons with LBP, a barrier to the widespread use of this methodology. In addition, the fear of participating in a high-load back muscle fatigue test [28,29] could result in aberrant patterns of muscle contraction [25]. To overcome these problems, our research group studied the SEMG data collected during dynamic back extensions performed at slow movement velocities and using a load equal to 50% MVC. This protocol complies with both evidence-based recommendations for resistance training in novice trainees [30,31], and the recommended level of muscle contraction to generate blood flow occlusion and muscle fatigue detectable using SEMG data [25]. By relying on ad hoc algorithms to analyze the time–frequency content of SEMG data collected during dynamic contractions [32,33], our research group demonstrated that this method may indicate neuromuscular aging processes in healthy pain-free volunteers [19]. However, given known alterations in trunk sensorimotor control and muscle morphology with LBP [10,34], it remains uncertain as to whether this novel test is marked by sufficient age-specific discriminatory sensitivity in cLBP persons. In fact, divergent findings on histomorphology (with a lower than expected content of glycolytic type II fibers in aging back muscles, and a higher than expected content of glycolytic type II fibers in cLBP) could support the theory that age-specific effects on the spectral content of SEMG data during a fatiguing contraction may be masked by cLBP [35,36,37,38]. In addition, the back extensors of healthy females and cLBP persons were found to be marked by a lower proportion of glycolytic fibers, and a higher resistance to fatigue than males [35,36,39,40,41,42]. This suggests that the aging-related effects on the back muscles observed during a dynamic contraction in individuals with cLBP, if existent, might differ between males and females.

This research sought to investigate, for the first time, if the IMDF-SEMG derived from recordings collected during a cyclic back extensor exercise protocol in exercise-naive persons would enable the detection of differences in back muscle function in younger vs. older male as well as female individuals with cLBP. In addition, the retest reliability of this method was investigated.

## 2. Materials and Methods

### 2.1. Ethics Statement

The Ethics Committee of the City of Vienna—Number: “EK 11-064-VK-NZ” approved the study protocol. All participants provided written informed consent, in accordance with the Declaration of Helsinki directives.

### 2.2. Participants

Study volunteers were recruited via word of mouth and by presenting the study to cLBP patients undergoing outpatient rehabilitation. All prospective participants received information that they would have access to six months of training at the Karl-Landsteiner Institute for Outpatient Rehabilitation Research after completion of the study.

A total of 294 persons affected by non-specific cLBP (159 females), between 18 and 90 years old, volunteered to participate in the study. They all completed a short screening questionnaire to assess the location, duration, and intensity of pain, as well as functional limitations and co-morbidities affecting study participants. Thereafter, eligible persons with cLBP completed a medical examination performed by a Physical and Rehabilitation Medicine specialist. Persons with cLBP were included in the study if they were otherwise healthy and suffered from LBP with a minimum of 30 mm on a visual–analogue scale (0–100 mm), as recorded during a 12 week period prior to screening. Patients were not eligible to participate in the study if the following conditions applied to them: (1) the receipt of health care advice for headache episodes and more than five episodes within the past year (one or more lasting more than two days), (2) neck pain equal to or exceeding 30 mm on a visual–analogue scale (0–100 mm), (3) headache within the last six weeks, (4) peripheral neurological deficit, (5) spinal fracture, (6) infection, (7) cancer, (8) previous surgery involving the back region, (9) previous experience with trunk muscle strength testing, (10) the performance of exercise more than two times per week or at a competitive level, (11) an inability to follow German verbal instructions, and (12) a BMI exceeding 35 kg/m². Participants were instructed not to take analgesic drugs, muscle relaxants, or psycho-pharmaceuticals within two days of testing. A total of 243 patients were included in the study.

### 2.3. Experimental Protocol

#### 2.3.1. Schedule of Assessments and Tasks

The schedule of assessments and tasks were similar to those described in a previous study conducted in healthy volunteers by our research group [18]. Participants completed five different tasks as follows: (1) basic anthropometric measurements and questionnaires to assess their motivation and physical activity level; (2) warm-up and isometric MVC of back extensors; (3) 20 min of rest, in which the SEMG electrodes were attached; (4) one sustained isometric back extension at 80% MVC for 30 s; and (5) after a resting interval of approximately 10 min, a cyclic back extension exercise (dynamic fatigue task) performed with a load equating to 50% MVC. All tests were guided by three experienced examiners (CS, MW, and RH) and a certified clinical psychologist.

The complete set of measurements was repeated in a second experimental session one to two days after the first, and further repeated in a third session approximately six weeks later.

#### 2.3.2. Equipment and Tests

##### Back Extension Dynamometer

The F110 back-extension device (DAVID^®^ health solutions, Helsinki, Finland) was used to assess maximum isometric back extension torque. The dynamometer is described elsewhere in detail [43], and consists of a “hip fixation mechanism” which integrates five components: adjustable footplates, adjustable knee pads, a pelvic belt, an adjustable seat, and a dorsal back pad. Participants were seated on the machine with their trunk flexed forward at 30°, and their arms at either side. As the dorsal back pad of the F110 device is necessary to minimize the risk of injury during free MVC trunk extension tests, but would likely interfere with the SEMG recordings from lumbar extensors, the “Total Trunk” (TechnoGym^®^, Cesena, Italy) exercise device was used to assess static and dynamic muscle fatigue. The TechnoGym^®^ device is built similarly to the F110 DAVID^®^ extension device, but it is equipped with a dorsal sacral pad instead of a back pad, and it therefore allows for unrestricted SEMG recording from the lower back.

##### Surface EMG

Active sensors with parallel-bar electrodes and triaxial accelerometers (Model Trigno, DelSys^®^, Boston, MA, USA) were used to collect SEMG data. After standard preparation of the skin, double-sided adhesive interfaces were used to attach the sensors. Accounting for the muscle fiber direction and according to the SENIAM guidelines and previous work [24,27,44], the SEMG sensors were positioned bilaterally at L5 (multifidus), L2 (longissimus dorsi), and L1 (iliocostalis lumborum). In positioning the EMG sensors, anatomical landmarks were used to confirm the proper positions of the sensors. The SEMG sensors were marked by a total effective gain of 909 V/V ± 5%, a bandwidth of 20–450 Hz, and a baseline noise <0.75 μV (RMS). SEMG data were sampled at 2 kHz using a 16-bit analog/digital (A/D) converter. The EMG Works^®^ software (DelSys^®^, Inc., Boston, MA, USA) was used for data collection.

##### Additional Accelerometer Data

A triaxial accelerometer (Trigno, DelSys Inc.^®^, Boston, MA, USA) attached to the dynamometer arm was used to monitor the trunk movements. Biomechanical variables (i.e., the range of trunk movement, and the peak and average movement velocities of the exercise bouts) were calculated using the accelerometer data. The triaxial accelerometer gathered data with a dynamic range of ±2.0 g, a maximum resolution of 0.016 g/bit, and a bandwidth of dc-50 Hz. The accelerometer data were sampled at 160 Hz with a resolution of 8 bits using the EMG Works^®^ software.

##### Other Tests

Hand grip strength testing: Following the manufacturer’s guidelines, study participants performed a series of three maximum grip strength tests (Jamar, Anaheim, CA, USA) in an unsupported upright seated position. The order was alternated between the right and left hand. A rest interval of 20 s followed each test. Tests were considered invalid and thus repeated if they differed more than 10%.

Questionnaires: During each session, participants rated their perceived back muscle fatigue level on an 11 point Borg visual–analogue scale with anchors 0 (no fatigue at all) and 10 (most severe fatigue ever experienced), and completed the International Physical Activity Questionnaire (IPAQ) [45], as well as health-related questionnaires. These were the Roland Morris disability questionnaire [46] and the avoidance endurance questionnaire [47].

#### 2.3.3. Test Procedures

##### Maximum Back Extension Test

In order to familiarize the participants with the equipment and the test procedures, they performed a warm-up exercise at a low load. Then, they completed two consecutive isometric MVC tests under supervision by an experimenter who provided instructions and encouragement in a standardized manner. Further trials were carried out until consistent data were collected. If the second MVC test varied by more than 10%, or if the peak effort was achieved later than 3 s after the onset of the contraction, the MVC test was repeated. The best out of two consistent trial scores was stored.

##### Cyclic Back Extension Exercise

Participants performed a static back extension at 80% MVC for 30 s with their trunks 30° ante-flexed and in a seated position. After resting for 10 min and performing a few trials without external load in order to familiarize themselves with the cyclic back exercise, participants were asked to perform 25 cyclic contractions with the device arm exerting a load equal to 50% MVC. Therefore, they repetitively flexed and extended their trunks between the upright sitting position and a 40° forward flexed position following the pace of a metronome and the verbal instructions provided by the experimenter. The movement velocity of the flexion–extension exercise bout was paced with a metronome set at 2 s intervals (2 s extension and 2 s flexion). If the participant was unable to perform 25 repetitions or the task-specific range of motion (ROM), or if the movement velocity could not be sustained, the test was stopped. If more than 15 consecutive repetitions were successfully performed, the data were considered suitable for analysis. Otherwise, the data were discarded. Immediately after the dynamic test, volunteers rated their perceived back muscle fatigue level using the BORG rating scale.

### 2.4. Signal Processing

MATLAB scripts (The MathWorks, Inc., Natick, MA, USA) were used to analyze the SEMG data. In order to avoid transitory behaviors, the first three seconds of the static tests and the first eight seconds (first two cycles) of the dynamic tests were discarded. The SEMG data were filtered using a 20 Hz high-pass and a 450 Hz low-pass Butterworth filter. From the second to the fifth second of the sustained 80% MVC contraction, the root-mean-square (RMS) values of the SEMG data were estimated for one-second intervals, as described in previous work [18].

**Figure 1:**
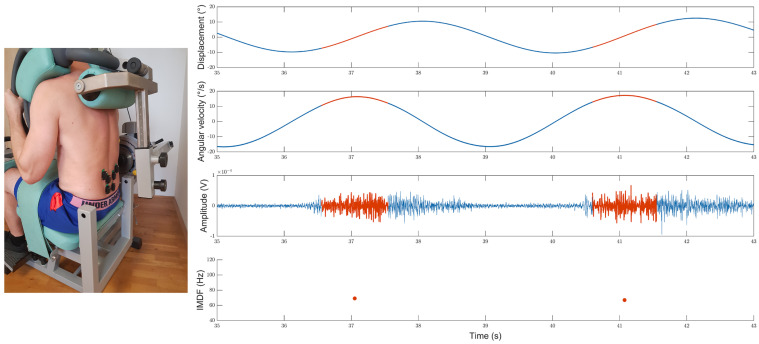
The picture on the left shows the positions of the SEMG sensors (Trigno, DelSys Inc.^®^, Boston, MA, USA) during the cyclic back exercise performed using the “Total Trunk” back exercise device (TechnoGym^®^, Italy). The plots on the right show the biomechanical and electromyographical data for a representative participant performing a cyclic back exercise bout. The upper graph provides the angular displacement (upper graph), the second graph the angular velocity, the third one the raw SEMG signal highlighting time intervals used for the time–frequency analysis, and the bottom graph the IMDF values derived from such time intervals. The concentric phase of the cyclic exercise is marked in red, the eccentric phase and turn phases in blue. For the bottom graph the red points represent the IMDF time series value generated by Cohen Class time-frequency representations for each SEMG sample.

For the concentric phases of the cyclic exercise, SEMG data segments were identified based on the accelerometer data monitored using the probe located on the arm of the Total Trunk device (Figure 1). The RMS and IMDF values were computed for all SEMG segments. 50% overlapping windows were used to derive multiple RMS and IMDF estimates from each data segment. The first and last 125 samples of the SEMG segments were discarded to avoid “edge” effects. IMDF calculations were based on Cohen class time–frequency representations [48] of the SEMG data. The algorithms used to derive the IMDF values [32] generated one median frequency value for each SEMG sample (i.e., an IMDF time-series).

Electromyographic representations of localized muscle fatigue were calculated from each series of 25 bursts of activity, by computing the least squares regression line of the IMDF estimates derived from each of the 25 EMG bursts of activity. The IMDF values of each exercise bout were averaged to obtain one IMDF value per cycle and phase. The slope of the regression line (calculated from these 25 values) served as the measure of localized muscle fatigue. The RMS values were normalized by the RMS value obtained during 4 s (i.e., seconds 3 to 7) of the 80% MVC test. A linear regression was derived from the RMS values, as described above. The regression line slope allowed us to estimate the rate of change in the RMS value of the SEMG data.

To account for inter-individual differences that would affect the spectral SEMG representations, such as subcutaneous tissue thickness, and following previous recommendations [49], both the RMS and the IMDF slopes were divided by their corresponding intercept values, resulting in normalized EMG indices of muscle fatigue. In order to improve the reliability, the normalized EMG fatigue parameter values for each of the three electrode pairs at the three lumbar levels (L1, L2, and L5) were averaged [27].

#### 2.4.1. Accelerometer Data Analysis

The trunk range of motion was monitored by utilizing the sagittal angular displacement data derived from a geometrical procedure, using gravity as reference, as described in previous work [19,50]. To calculate individual angles, the accelerometer data collected using all the probes were combined (Figure 1).

### 2.5. Definition of Variables

The following dependent variables were used in the analysis: MVC (trunk extensor strength), mean IMDF-SEMG onset values calculated from the linear regressions, and mean IMDF-slopes normalized using the IMDF-SEMG initial values. The independent variables were subjects’ age (older or younger than 50 years of age), sex, and test day (3 days). The R software environment for statistical computing was used for all statistical analyses [51].

### 2.6. Sample Size Estimation

The sample size for the mixed ANOVA [52] was estimated via Monte Carlo power simulations. These simulations considered all of the age- and sex-specific effect sizes previously observed from a similar study by our research group [19], the differences in the psycho-emotional features of cLBP participants that might affect their willingness and motivation to provide their best back extension performance possible [49], a total of five comparisons at an alpha of 0.01, a power of 0.90 (1-beta), and a loss of 10% of SEMG signals due to recording artefacts. Investigating a total sample of 240 participants with cLBP was deemed necessary to detect small to medium age and sex-dependent effects between groups. Figure S1 provides a graphic illustration of the simulations, considering different effect sizes.

### 2.7. Age- and Sex-Specific Subgroups

For the main analyses that sought to identify age- and gender-specific differences, artefact-free SEMG data from the full set of electrodes derived from biomechanically comparable (velocity and acceleration) cyclic exercise tasks were available for 85 older (48 women) and 92 younger (39 women) participants. These data were selected to control for any confounders that could occur from group imbalances in the biomechanics of task performance or due to missing EMG variables. Our primary analysis was performed with the fitted data of the missing EMG variables of individual electrodes. The fitting of data could be achieved if the SEMG recordings of one of the two (paired) electrodes per segment were artifact free. Then, the artifact-free SEMG data were duplicated, and both the onset and the slope values were corrected using the left–right imbalance ratios of the respective lumbar segment obtained from the 177 LBP individuals with full datasets. Hence, data from a total of 108 older (60 women) and 114 younger (52 women) individuals with LBP were selected for analysis.

Descriptive statistics was used to summarize the participants’ characteristics. A mixed effects model with fixed effects for “age”, “sex”, and “test number”, and random effects for “persons” were computed for each outcome variable, in order to evaluate age-specific and sex-specific between-group effects. As in [53], *p*-values were Bonferroni corrected and considered as significant if *p* ≤ 0.01. If significant effects for “age”, “sex”, or their respective interactions were detected, post hoc analyses were conducted using estimated marginal means (EMMs) [54]. Residual diagnostics allowed us to validate the mixed effects models. Cohen’s d effect sizes were also calculated, and values of 0.2, 0.5, and 0.8 were used for small, medium, and large effect sizes, respectively.

Linear mixed models were used to test whether the variables test day, age, sex, pain intensity, physical activity behavior scores (IPAQ), AEQ-derived movement behavior, and disability score (RMDQ, PDI) affected the normalized IMDF fatigue slopes of each of the different electrode-recording sites.

### 2.8. Reliability of SEMG Measures

The Generalizability Theory (G-Theory) [55,56,57] was used to examine the retest reliability of the SEMG-based spectral measures. Both the absolute standard error of measurement (SEM) and the coefficients of dependability (D), the latter equating the Intra Class Correlation Coefficient (ICC), were derived from a “multi-factorial random-effects Analysis of Variance (ANOVA)” model that included several sources of measurement error (related to subject, day, side, subject × day, and subject × side). Note that the G-theory distinguishes between absolute and relative decisions: absolute decisions are criterion-referenced and they occur if the subject’s measurement results are independent of the performances of other subjects.

## 3. Results

**Figure 2:**
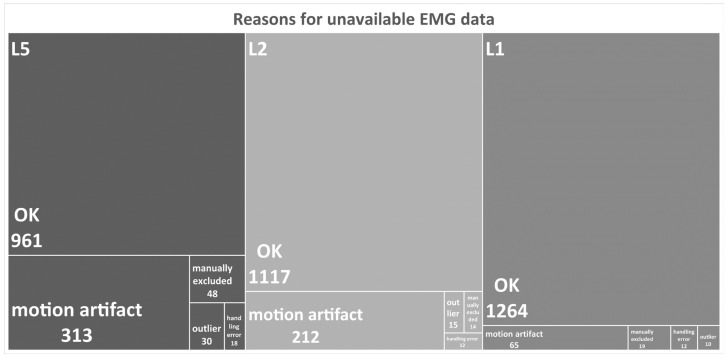
Main reasons for excluding SEMG signals from the analysis. Note that the EMG recordings were from three pairs of electrodes, and that the experiments were performed on three different days.

A total of 177 out of the 243 LBP study participants showed artefact-free SEMG data for all six electrode-recording sites. The EMG recordings of 21 participants were not considered in the analysis, either because the data displayed biomechanical inconsistences when performing the exercise, or because the participants were unable to complete the task. The data of an additional 45 participants had artefacts affecting the SEMG data because of intermittent electrode detachment during the performance of the cyclic exercise; filtering significantly attenuated these artifacts. Overall, the recordings from 222 individuals were considered for the analyses herein presented. The reasons for the exclusion of individual SEMG signal recordings are summarized in Figure 2.

For the study participants whose data were included in the analyses, all the values of the demographic variables (“age”, “height”, and “weight”), the functional scores (“maximum back extension strength” and “hand grip strength”), and the fatigue ratings at the end of the exercise task did not differ from those whose data had to be excluded from further analysis (as described above).

All the other variables (i.e., BMI, maximum handgrip strength score, perceived back muscle fatigue ratings at the end of the cyclic exercise, and RMDQ-PDI scores) revealed age- and/or sex-specific between-group differences (Table 1).

The biomechanical performance quality variables “trunk range of motion”, “peak movement velocity”, and “mean movement velocity” for the concentric portion of the cyclic back extension exercise were comparable between the age and sex subgroups. It is worth noticing that these biomechanical variables were approximately constant throughout the dynamic exercise in each of the two age and sex subgroups (Table 1).

### 3.1. Age-Dependent IMDF-SEMG Changes during the Cyclic Exercise

The IMDF-SEMG slope values (derived from the IMDF-SEMG data normalized by the onset value) demonstrated a significant shift toward the lower frequencies of the SEMG data collected during the exercise test, in all electrode sites. These slopes were generally greater in younger than older participants, and in males than females. A significant age- and sex-specific interaction, accompanied by significant age- and sex-specific effects, was observed for the L2 electrodes. A Post hoc analyses of this electrode site revealed significantly steeper slopes in younger vs. older male participants (*p* < 0.001), and in younger males vs. females (*p* < 0.001).

Significant age-specific as well as sex-specific effects were observed for all electrodes (taken all together). The L5 electrode-recording site and the L2 electrode-recording site displayed the most negative slope values. Post hoc analyses revealed a larger decrease during the exercise in IMDF-SEMG values, in younger vs. older male participants, and in younger male vs. female ones for all electrodes (*p* ≤ 0.01). The comparison of the results for younger and older women showed significantly steeper slopes for the L5 recording site. The imbalance scores were comparable in younger and older participants, as well as in male and female CLBP participants. The factor repetition of test days revealed a significant effect on the data collected from L1 between test sessions one and two, and in the data collected from L2, and for all electrodes between test sessions two and three. The results showing age- and sex-specific differences in the IMDF-SEMG data are summarized in Table 2, and those of the post hoc analyses are summarized in Table 3.

### 3.2. Retest Reliability of the IMDF-SEMG Estimates in Older and Younger Individuals

The absolute standard error of measurement (SEM) values derived from the IMDF-SEMG onset value estimates were found to be the highest for L5 and L2, and the lowest for the recording site associated with the most negative IMDF-SEMG slope. The absolute SEM values of the normalized IMDF-SEMG slopes were the largest for the recording site associated with the most negative IMDF-SEMG slope, and the smallest for L1. No major differences were observed between the two age subgroups. D-values for the IMDF-SEMG onset value estimates ranged between 0.72 and 0.84 for all the different recording sites, indicating good to excellent reliability. The corresponding D-values of the normalized IMDF-SEMG slope values ranged between 0.4 (L1) and 0.71. These D-values were comparable in younger and older cLBP individuals. Table 4 shows all the ICC and absolute SEM values observed for the different SEMG recording sites.

## 4. Discussion

In our quest for a predictive, digital biomarker that could detect early signs of neuromuscular aging, we investigated whether parameters derived from the time–frequency representation of SEMG data could identify age-specific differences in individuals with cLBP who engaged in a moderately fatiguing cyclic back extension exercise. The main findings from the comparison of the data collected from a group of younger individuals and from older adults with cLBP were:

1. Normalized IMDF-SEMG fatigue slope values were significantly smaller in older than younger individuals with cLBP, with differences that were more prominent in males than in females;
2. Normalized IMDF-SEMG fatigue values were greater in younger male participants with cLBP vs. female participants with cLBP;
3. Neither pain intensity nor health-related disablement had a major effect on the IMDF-SEMG slope;
4. The relative reliability was overall moderate-to-good for the IMDF-SEMG slope values (derived from data normalized by the IMDF-SEMG onset value); the absolute reliability indicated a small test–retest variability for both the IMDF-SEMG slope values and the IMDF-SEMG onset values.

### 4.1. Methodological Considerations, Individuals’ Maximum Back Extension Strength, and Mechanical Performance Parameters during the Cyclic Exercise

A reliable interpretation of the SEMG variables depends on carefully controlling the level of generated force output (which was determined to be relative to each individual’s MVC), as well as controlling biomechanical performance variables [25,58]. Maximum back extension scores in the younger study participants with cLBP were similar to those observed from healthy, pain-free males and females who had undergone testing using the same protocol and similar equipment [43]. The back extension strength scores in our older participants with cLBP were, despite significantly lower handgrip strength scores, comparable with those observed from their younger counterparts, suggesting that the older participants were able to produce true or close to true maximum scores. The fact that comparable high maximum back strength scores were observed likely indicates that fear-related avoidance behaviors or associated psychological issues [28,29] neither affected the maximum performance measures collected in the study nor the load equivalence in the two age groups.

The biomechanical variables “range of motion” and “mean/peak movement velocity” were comparable between groups, and were approximately constant throughout the exercise. This is evidence that the overall biomechanical quality of the exercise was high, and that confounders potentially affecting the SEMG recordings due to the variability in biomechanical performance of the cyclic exercise unlikely affected the between-group comparisons of the SEMG-based parameters.

At the end of the cyclic exercise, older participants reported significantly less back muscle fatigue than younger participants. This was observed even though the corresponding rating scores still indicated the fatigue to be moderate, and the work performed during the exercise was comparable between the two age groups. Such observations are consistent with previous research that found that muscle endurance during sustained and slow dynamic contractions is better preserved in older than younger healthy individuals [59,60,61]. In addition, findings from a recent study conducted in young healthy individuals appear to externally validate the fatigue ratings observed amongst the younger participants in our study [49]. In this previous research, participants were able to perform a maximum of 34 cycles of back extensions on a Roman chair. The back load used was similar to that in our study, and the cyclic exercise was performed within the same range of movement and at a comparable movement velocity [49].

### 4.2. IMDF-SEMG Characteristics

The significant decrease in the IMDF-SEMG values for all the electrode sites was associated with RMS-SEMG onset values that exceeded those of the 80% MVC test by 20% to 30% for most of the electrode sites (please see the normalized RMS-SEMG data in Table S1). As motor unit recruitment is likely complete, even in those muscles with the largest recruitment threshold at 80% MVC, the high RMS-SEMG values observed for the concentric phases of the exercise suggest that most or all recruitable motor units within the detection volume of the surface electrodes used in the study were active and/or entrained at higher firing rates than during the 80% MVC static test [62]. Lower recruitment thresholds of motor units with more motor units firing simultaneously at a comparable submaximum force were observed in older than younger persons [63]. This suggests that the motor unit recruitment of back extensors during the exercise was more complete in the older than in the younger participants. If so, this would have challenged the higher threshold motor units in our older participants to a larger extent than in our younger ones, and consequently should have induced more IMDF-SEMG back muscle fatigue in older than in younger participants, which was not the case. It is worth noticing that the time-dependent decrease in SEMG would more likely result from changes in electromyographic behavior within the glycolytic muscle fibers. These glycolytic fibers (type II and glycolytic type I fibers) compose the higher threshold motor units [20,21,24], which are recruited later in static or slow concentric contractions of increasing force [23]. As potential biomechanical confounders of the SEMG parameters appeared to be negligible in this study, and other factors that could affect the SEMG signal over time appeared to be similar between groups, the age-related differences in the IMDF-SEMG decline values observed during the exercise at L5 and L2, and the electrode site associated with the most negative slope of the IMDF time-course in participants with LBP of this study likely related to differences in the availability and/or behavior of the active electromyographically fatiguing glycolytic muscle fibers [23,24,64,65]. Hence, our test might detect neuromuscular aging processes that are mediated by a decrease in the pool of available glycolytic muscles fibers in the back extensors. Noteworthily, this would be the case, despite the presence of muscle structural and metabolic processes that could induce more spectral SEMG fatigue in persons with cLBP, and which would counter the age effects observed in our study [35,36,37,41]. This observation is in line with the previous findings of our research group pertaining to the use of frequency parameters derived from SEMG data showing their potential use as a muscle-age-specific diagnostic biomarker in healthy, pain-free individuals, or in persons with cLBP [18,19,66].

The observation of significant age effects in the spectral SEMG parameters appears to contrast with those of a recent study that examined age-specific differences in MDF-SEMG parameters derived from data collected from the back extensor muscles in healthy volunteers or in persons with cLBP [66]. This study induced back muscle fatigue via sustained back extensions at 50% MVC with participants positioned in a Roman chair in a semi-flexed, prone lying position, and with shanks in a fix position. As the biceps femoris, hip, and back extensors are tightly coupled via the fascia thoracolumbalis and ligamentum sacro-tuberale, and as they contribute to the generation of trunk extension torque [67,68], Roman chair exercises may allow for a greater variability in motor control strategies than the test devices used in our study. Consequently, variable, non-predictable muscle synergies involving muscles other than the back extensors could contribute to the biomechanical performance of fatiguing Roman chair exercises, thereby preventing the detection of aging processes when using the spectral parameters of the SEMG data. Testing individuals in a seated position with the hips and knees flexed at approximately 95 to 100 degrees, using the constraints of the TECHNOGYM or the DAVID device, appears to challenge the back extensor muscles to a greater extent than the Roman chair fatigue-based test [58], and thus allow for more consistent results.

Our observations of less clearly pronounced age-related IMDF-SEMG fatigue effects in females than males with cLBP suggest that spectral SEMG parameters derived by relying on moderately fatiguing exercise protocols might be more effective in detecting age-related neuromuscular differences in males than females with cLBP. Conversely, previous findings derived from analyses of SEMG data recorded during 80% MVC static, sustained back extensions showed age-related spectral SEMG fatigue effects more prominently in females than in males with cLBP [66]. This suggests that age effects on the spectral SEMG compression toward the lower frequency range observed during the cyclic back extension exercise at 50% of the maximum back extension strength could have been confounded by sex-specific differences in blood flow occlusion to the back muscles. This observation is supported by evidence demonstrating less oxygen provision to the muscles of male than female individuals during submaximal contractions at force output levels that are matched relative to the maximum force output [42,59]. A diminished rate of oxygen delivery in males would stimulate more glycolytic muscle metabolic activity, as well as impairing the muscle force generating capacity of oxidative metabolic fibers [69]. In addition, cumulating lactate, the predominant factor causing a pH decrease, which would slow muscle fiber conduction velocity and alter the shape of the motor unit action potentials, would decrease SEMG fatigue-related spectral compression [20,21,70]. The lower sensitivity to the age-related phenomena of spectral SEMG measures recorded from a moderately demanding cyclic back extension exercise in females with cLBP compared to males with cLBP could also be the result of smaller size, fast fatiguing, high threshold back muscle motor units in females than males [36]. However, further research is warranted to determine the optimal level of muscle cyclic back extension contraction to be used in tests in males vs. females to detect age-related changes.

Previous research has found that the multi-segmental paravertebral back extensors located in the intermediate column, such as the iliocostalis lumborum muscle, contain a twofold higher number of muscle spindles than those in the medium column (longissimus dorsi, at L2) or the deep mono- to oligo-segmental back muscles (multifidus, at L5), which appeared to have almost no spindles [71]. Given that motor unit recruitment relates to the number of muscle spindles, one would anticipate that the recruitment of motor units in the medial back extensors (multifidus at L5 and longissimus dorsi at L2) would be completed at 40% to 50% MVC force levels. In contrast, the recruitment threshold in multi-segmental muscles would be higher and completed at 70% to 80% MVC [72]. As major segmental spine stabilizers, the multifidus at L5 and the longissimus dorsi at L2 may be assumed to be biomechanically more involved in the cyclic back extension test compared to the iliocostalis lumborum at L1 [73]. Thus, one would expect a higher level of recruitment and higher firing rates in the multifidus and longissimus dorsi muscles compared to the iliocostalis lumborum. Consequently, a smaller proportion of higher threshold, glycolytic motor units may be activated or firing at lower rates in the iliocostalis lumborum muscle at L1 compared to the multifidus at L5 and the longissimus dorsi at L2. It follows that, in agreement with our findings, one would anticipate a more modest change in the SEMG spectral parameters enabling the detection of age-related effects for the iliocostalis lumborum muscle at L1 than for the multifidus at L5 and the longissimus dorsi at L2. This observation suggests that the L1 electrode site is not as suitable for the detection of the aging effects on muscles.

### 4.3. Reliability of SEMG Variables

This study further sought to investigate the day-to-day retest reproducibility of SEMG fatigue-related variables. Depending on the recording site, indicators suggest a moderate-to-good relative reliability for the spectral SEMG variables. However, absolute reliability was found to be highly variable for both the normalized slopes and the SEMG onset values. This was observed for both age-specific subgroups. If one compares the results of the current study with those of earlier research in which healthy individuals performed cyclic free lifting exercises [19], or where individuals with cLBP performed a sustained 80% MVC back extension [66], it is apparent that the reliability scores of this study were lower. Future research will need to optimize the test protocols in order to improve the precision of the IMDF-SEMG estimates (Figure S2), and/or to introduce new IMDF-based variables, if the dynamic SEMG back extension test is further developed to derive digital biomarkers that are suitable for detecting early signs of back muscle sarcopenia.

### 4.4. Limitations

One may argue that other factors than those related to the slowing in muscle fiber conduction velocity affected the age- and sex-specific differences in SEMG spectral compression observed during the cyclic exercise. In fact, changes in motor unit firing statistics with fatigue, and their correlation to each other, as well as changes in the spatial distribution of activated motor units, could affect the spectral-related fatigue changes over time. Regarding the firing statistics, the RMS-SEMG increase observed in this study was similar in older and younger participants, as well as in males and females. Thus, an increase in firing rate and/or the recruitment of additional motor units during the exercise would not explain the differences in the SEMG spectral compression. Considering that muscle fatigue may influence common drive, as demonstrated recently in intermittent, fatiguing 20% isometric knee extensions [74], an increase in cross-correlation could likely increase the SEMG spectral fatigue slope in younger persons to a higher degree than in older ones. Such an assumption would be based on research that found firing rate fluctuations between pairs of motor unit action potential trains to display less correlation (common drive) in older than in younger muscles [63]. Such a correlation would not be expected to increase with the duration of the exercise in older participants, who demonstrate a lack in common drive. The relevance of this confounder may be of minor relevance, as the sex-specific IMDF- SEMG differences observed would be expected to predominantly relate to SEMG fatigue-induced changes in motor unit action potential shape and firing rate, rather than to differences in common drive between males and females of the same age.

Other confounders of the SEMG spectral variables, such as non-travelling potentials and scattered innervation zones, most likely equally affect the data collected from the two groups [75,76], and thus are unlikely to have a major significant effect on the between-group differences. Furthermore, needle electromyographic signs of muscle denervation i.e., fibrillation potentials (and positive sharp waves) which frequently present in older back muscles are unlikely detected by the SEMG sensors that we used in this study. Even, if these potentials were theoretically represented within the SEMG spectrum, these would not contribute to age-related spectral SEMG fatigue differences as their shape remains stable over time. In addition, signal-to-noise ratios may have differed due to differences in subcutaneous tissue thickness in the back, and affect IMDF onset values. However, such a confounder was minimized by normalizing the IMDF slopes by the SEMG onset values [23].

We attempted to record a full set of SEMG data during exercise testing from all 243 participants. We were successful in obtaining artefact-free recordings from all electrode sites in 177 individuals. One of the main reasons leading to SEMG artefacts was the intermittent loss of electrode-skin contact, which occurred most frequently for recording sites at L5 and L2. As such a loss of electrode recordings may have reduced the statistical power for detecting age-specific differences, we decided to analyze the fitted SEMG data. Findings from the analyses of the fitted data and those of the 177 participants with artefact-free EMG recordings were similar. Future research will have to address the electrode detachment problem when a cyclic exercise is to be performed.

### 4.5. Implications

Monitoring the spectral compression of the SEMG data recorded from back muscles during a cyclic exercise at moderate efforts would allow one to detect the early signs of an accelerated neuromuscular aging process, particularly in males, and to a lower extent, in females with LBP. Notably, the dynamic test used in this study does not allow one to identify the specific underlying causes of the altered IMDF-SEMG fatigue-related behavior in older patients with cLBP. This could result from either the long-term-recruitment failure of innervated high threshold type II glycolytic muscle fibers [77], or from non-recruitable, denervated muscle fibers due to axonal degeneration affecting type II fibers because of neuromuscular regeneration, or from the entrapment neuropathy of the dorsal medial branch of the spinal nerve [78]. Recent research investigating the normative values for paraspinal denervation using needle EMG found older asymptomatic study participants with more denervation than younger ones [79].

## 5. Conclusions

As in healthy, pain-free individuals, the observation of trends in IMDF-SEMG values during a dynamic test proved to be a successful approach for reliably distinguishing between back muscle function in younger vs. older men, and to a smaller extent, women with cLBP. These findings provide motivation for further developing this approach into a screening test that would be readily available in the clinic, and intended to early detect aging back muscles prior to more obvious age-related muscle function changes typically observed in the target population. The early detection of aging back muscles would be relevant to the design of therapeutic interventions that are intended to prevent an early aging process and disability in older individuals.

## Figures and Tables

**Table 1:** Demographic variables and variables related to the biomechanics of test performance. *p*-values are provided for differences between age- and sex-specific groups. *p*-values were adjusted for multiple comparisons (age- and sex-specific effects); *p* < 0.025 was considered significant. RMDQ = Roland Morris Disability Questionnaire; IPAQ = International Physical Activity Questionnaire; AEQ = Avoidance Endurance Questionnaire; FAR = Fear avoidance response; ER = Endurance response; VAS = Visual Analogue Scale; ROM = Range of Motion; <50 vs. >50 = participants’ age groups (in years); SE = Standard error.

	<50 Years Mean (SE)	>50 Years Mean (SE)	<50 vs. >50 *	Males Mean (SE)	Females Mean (SE)	Males vs. Females *
Age (years)	34.0 (1.3)	64.1 (1.1)	<0.001	47.6 (2.3)	50.5 (2.2)	0.37
BMI (kg/m²)	24.48 (0.42)	26.80 (0.41)	<0.001	26.33 (0.35)	24.72 (0.53)	0.012
Back Extension Strength (Nm)	209.1 (9.2)	192.6 (9.9)	0.22	265.3 (7.1)	148.3 (4.8)	<0.001
Grip Strength (kg)	39.3 (1.6)	31.3 (1.5)	<0.001	46.9 (1.0)	25.9 (0.6)	<0.001
Back Muscle Fatigue (VAS 0–10 (strongest))	5.6 (0.3)	4.5 (0.3)	0.004	5.7 (0.3)	4.5 (0.3)	0.001
Pain (last month) (VAS 0–10 (worst))	48.9 (1.3)	52.1 (1.9)	0.15	49.9 (1.5)	50.3 (1.3)	0.84
Pain (test day) (VAS 0–10 (worst))	37.2 (1.1)	39.9 (1.5)	0.15	37.8 (1.2)	39.1 (1.3)	0.45
RMDQ (0–24 (worst))	5.54 (0.37)	8.37 (0.43)	<0.001	7.25 (0.31)	6.22 (0.46)	<0.001
IPAQ (kcal/wk)	4141 (484)	4306 (330)	0.78	4049 (364)	4417 (414)	0.50
AEQ-FAR (0–6 (strongest))	1.53 (0.09)	1.70 (0.09)	0.16	1.69 (0.08)	1.52 (0.10)	0.19
AEQ-ER (0–6 (strongest))	3.18 (0.08)	3.54 (0.07)	0.002	3.30 (0.08)	3.44 (0.09)	0.24
Exercise Velocity (con °/s)	33.2 (1.2)	33.2 (0.6)	0.95	33.2 (1.2)	33.2 (1.2)	0.99
Exercise Velocity (con changes ^1^ °/s)	0.02 (0.002)	0.01 (0.001)	0.14	0.02 (0.002)	0.02 (0.002)	0.23
ROM (°)	33.8 (0.6)	33.2 (0.6)	0.60	32.7 (0.6)	33.8 (0.6)	0.20
ROM changes ^1^ (10^−3^)°	0.26 (0.02)	0.22 (0.02)	0.045	0.22 (0.02)	0.25 (0.02)	0.22

* *p*-value; ^1^ changes due to fatigue.

**Table 2:** Results of the IMDF-SEMG analysis from 221 individuals with cLBP who performed the cyclic submaximum exercise. For these 221 persons, a fitted EMG dataset was available for at least one test day, and the biomechanical variables of test performance were comparable between age groups. Note that *p*-values were adjusted for multiple comparisons (3 electrode pairs and 5 comparisons) using a Bonferroni correction. A *p* < 0.01 was considered significant. Please also note that changes in the IMDF-SEMG value during the exercise (normalized by the onset value) were significant, with *p* < 0.0025, and they are herein marked by “*”. L5 refers to the multifidus, L2 to the longissimus, and L1 to the iliocostalis muscle recording sites.

	Mean (SE)				Linear Mixed Effects Model							
Electrode Level	<50 Years (n = 114)	>50 Years (n = 108)	Males (n = 110)	Females (n = 112)	Age *t*; *p*	Age d	Sex *t*; *p*	Sex d	Age × Sex *t*; *p*	Age × Sex d	Day 1 vs. 2 *t*; *p*	Day 1 vs. 3 *t*; *p*
Onsets												
All	63.1 (0.8)	62.6 (0.8)	61.0 (0.8)	64.6 (0.8)	−2.78; 0.003	0.37	−0.88; 0.38	0.12	3.37; 0.001	0.45	0.16; 0.86	−0.46; 0.67
L5	72.7 (1.2)	74.9 (1.3)	71.0 (1.2)	76.3 (1.2)	−1.32; 0.16	0.18	−0.46; 0.65	0.06	2.58; 0.008	0.35	−0.38; 0.69	−1.44; 0.17
L2	62.0 (0.9)	60.5 (1.0)	59.1 (0.9)	63.4 (1.0)	−3.52; <0.001	0.47	−0.91; 0.37	0.12	3.76; <0.001	0.50	−0.90; 0.35	1.19; 0.25
L1	54.6 (0.6)	52.5 (0.6)	53.0 (0.7)	54.2 (0.6)	−3.36; 0.001	0.45	−1.26; 0.20	0.17	2.86; 0.003	0.39	−0.22; 0.83	−0.68; 0.50
m.n	49.4 (0.6)	48.2 (0.6)	48.0 (0.6)	49.6 (0.6)	−3.23; 0.001	0.43	−1.13; 0.26	0.15	3.24; 0.001	0.43	−0.70; 0.47	−0.58; 0.56
Changes normalized to Onsets												
All	−0.13 (0.01) *	−0.07 (0.01) *	−0.13 (0.01) *	−0.08 (0.01) *	4.67; <0.001	0.62	3.96; <0.001	0.54	−1.98; 0.043	0.27	−2.10; 0.025	−3.03; 0.004
L5	−0.18 (0.01) *	−0.10 (0.01) *	−0.18 (0.01) *	−0.11 (0.01) *	3.43; <0.001	0.46	3.48; <0.001	0.48	−0.79; 0.42	0.11	−1.05; 0.29	−1.21; 0.25
L2	−0.14 (0.01) *	−0.06 (0.01) *	−0.13 (0.01) *	−0.08 (0.01) *	5.73; <0.001	0.78	4.47; <0.001	0.62	−3.44; <0.001	0.47	−1.44; 0.14	−3.76; 0.001
L1	−0.08 (0.01) *	−0.05 (0.01) *	−0.08 (0.01) *	−0.05 (0.01) *	2.42; 0.013	0.33	1.74; 0.11	0.24	−0.70; 0.47	0.10	−2.53; 0.008	−2.14; 0.040
m.n.	−0.27 (0.01) *	−0.21 (0.01) *	−0.28 (0.01) *	−0.21 (0.01) *	4.34; <0.001	0.58	4.57; <0.001	0.62	−1.84; 0.062	0.25	−1.56; 0.11	−2.00; 0.051
un.imb.	10.6 (0.51)	11.4 (0.8)	10.42 (0.48)	11.48 (0.79)	0.03; 0.98	0.00	0.24; 0.78	0.03	0.52; 0.59	0.07	1.07; 0.34	0.26; 0.73
c.imb.	−5.2 (0.63)	−4.36 (1.0)	−4.45 (0.66)	−5.12 (0.9)	0.34; 0.72	0.05	−0.65; 0.41	0.09	0.32; 0.75	0.05	0.27; 0.80	−0.24; 0.77

m.n. = most negative electrode; un.imb. = uncompensated imbalances; c.imb. = compensated imbalances; All = all electrode-recording sites (L5, L2, and L1 pooled); <50 years; >50 years = participants’ age groups (in years); SE = Standard error; *p* = *p*-value; *t* = *t*-statistic; d = Cohen’s d; n = number of participants for whom a full set of electrode recordings was available; * = significant change (*p* < 0.0025 = Bonferroni-corrected significance level for the 5 levels and 4 subgroups = 0.05/20).

**Table 3:** Results of the post hoc analyses of the IMDF-SEMG fatigue changes (normalized by the onsets value) using estimated marginal means (EMMs) derived from the different electrode pair recording sites, from all electrodes, and the electrode demonstrating the most negative MF-SEMG slope. Note that *p*-values were adjusted for multiple comparisons (3 electrode pairs and 5 comparisons) using a Bonferroni correction; *p*< 0.01 was considered significant. <50 vs. >50 = participants’ age in years; SE = Standard error.

Electrode Level	Sex	Difference Mean (SE)	<50 vs. >50 *p*-Value	Age	Difference Mean (SE)	Males vs. Females *p*-Value
All electrodes	Male	−0.08 (0.02)	<0.001	<50 years	−0.07 (0.02)	<0.001
	Female	−0.03 (0.02)	0.05	>50 years	−0.02 (0.02)	0.3
L5 multifidus	Male	−0.08 (0.02)	0.001	<50 years	−0.08 (0.02)	0.001
	Female	−0.06 (0.02)	0.002	>50 years	−0.06 (0.03)	0.03
L2 longissimus	Male	−0.12 (0.02)	<0.001	<50 years	−0.09 (0.02)	<0.001
	Female	−0.02 (0.02)	0.3	>50 years	0.01 (0.02)	0.6
L1 iliocostalis	Male	−0.04 (0.02)	0.016	<50 years	−0.03 (0.02)	0.083
	Female	−0.02 (0.02)	0.1	>50 years	−0.01 (0.02)	0.5
Most negative IDMF fatigue	Male	−0.09 (0.02)	<0.001	<50 years	−0.09 (0.02)	<0.001
	Female	−0.04 (0.02)	0.07	>50 years	−0.04 (0.02)	0.07

**Table 4:** Test–retest reliability of the main outcome variables derived from the IMDF-SEMG recorded on three different test days. D = coefficient of dependability, equating the Intra Class Correlation Coefficient; SEM = Standard Error of Measurement; <50, >50 = two age groups.

	IMDF-SEMG			
Electrode Level/Age Group	Onsets		Changes Normalized to Onsets	
	D1	SEM	D ^1^	SEM
all electrodes	0.77	5.45	0.62	0.06
<50 years old	0.80	5.14	0.66	0.06
>50 years old	0.75	5.76	0.46	0.06
L5	0.84	6.31	0.67	0.08
<50 years old	0.85	6.12	0.71	0.07
>50 years old	0.82	6.51	0.53	0.08
L2	0.84	5.07	0.47	0.09
<50 years old	0.82	5.21	0.54	0.09
>50 years old	0.85	4.92	0.19	0.09
L1	0.76	4.15	0.38	0.07
<50 years old	0.74	4.40	0.43	0.08
>50 years old	0.78	3.88	0.16	0.07
most negative	0.72	4.36	0.71	0.06
<50 years old	0.74	4.20	0.75	0.06
>50 years old	0.70	4.54	0.53	0.06

^1^ D = D-value.

## Data Availability

The raw data are available upon request. Authors ensure appropriate measures are taken so that raw data is retained in full for a reasonable time after publication.

## Supplementary Materials

The following supporting information can be downloaded at: https://www.mdpi.com/article/10.3390/bioengineering10030300/s1; Table S1: RMS-EMG values; Figure S1: Monte Carlo simulations; Figure S2: electrode sites marked by most negative IMDF-EMG slope.
