# Supplementary material for: The Back Muscle Surface Electromyography-Based Fatigue Index: A Digital Biomarker of Human Neuromuscular Aging?
Source: Bioengineering (Basel). 2023 Feb 27;10(3):300. doi: 10.3390/bioengineering10030300 (PMC10045844; doi:10.3390/bioengineering10030300)

Table suppl S1. Results of the RMS-EMG from 221 individuals with cLBP who performed the cyclic submaximum back exercise. For these 221 persons a fitted EMG dataset was available on at least one test day, and the biomechanical variables of test performance were comparable between age groups. Note that P-values were adjusted for multiple comparisons (3 electrode pairs, 5 comparisons) using Bonferroni correction. A p< 0.01 was considered significant. Please also note that changes of the RMS-SEMG value during the exercise and normalized by the onset value are significant with p<0.0025 and herein marked by "*". L5 refers to the multifidus, L2 to the longissimus, and L1 to the iliocostalis recording sites.

	**Mean (SE)**				**Linear mixed effects model**							
**Electrode level**	**<50yrs**	**>50yrs**	**Males**	**Females**	**age t; p**	**age d**	**sex t; p**	**sex d**	**ageXsex t; p**	**ageXsex d**	**day1vs2 t; p**	day1vs3 t; p
Onsets												
All	1.3 (0.02)	1.3 (0.04)	1.28 (0.02)	1.23 (0.04)	0.75; 0.34	0.10	-0.34; 0.68	0.05	-0.61; 0.53	0.08	-2.24; 0.045	0.23; 0.70
L5	1.3 (0.03)	1.2 (0.11)	1.30 (0.03)	1.13 (0.11)	0.10; 0.83	0.01	-0.59; 0.22	0.08	-0.56; 0.54	0.07	-1.68; 0.13	0.24; 0.63
L2	1.3 (0.02)	1.3 (0.04)	1.32 (0.03)	1.30 (0.04)	1.33; 0.13	0.18	0.08; 0.93	0.01	-0.69; 0.49	0.10	-1.87; 0.10	-1.35; 0.19
L1	1.2 (0.02)	1.3 (0.02)	1.23 (0.02)	1.25 (0.02)	0.62; 0.53	0.08	0.20; 0.85	0.03	0.42; 0.68	0.06	-1.32; 0.18	1.75; 0.08
m.n.	0.9 (0.02)	0.8 (0.21)	0.97 (0.02)	0.73 (0.20)	0.21; 0.24	0.02	-0.07; 0.71	0.01	-1.06; 0.37	0.09	-1.06; 0.37	0.11; 0.49
Changes normalized to Onsets												
All	0.23 (0.03)*	0.13 (0.02)*	0.23 (0.03)*	0.14 (0.02)*	-2.06; 0.043	0.27	-1.91; 0.07	0.26	0.62; 0.52	0.08	1.14; 0.25	2.39; 0.009
L5	0.20 (0.03)*	0.08 (0.05)	0.20 (0.03)*	0.09 (0.05)	-1.07; 0.14	0.14	-0.94; 0.21	0.12	-0.35; 0.71	0.05	-0.24; 0.83	1.13; 0.047
L2	0.23 (0.03)*	0.15 (0.03)*	0.23 (0.03)*	0.15 (0.02)*	-2.28; 0.032	0.31	-2.18; 0.040	0.30	1.55; 0.11	0.21	1.46; 0.08	2.52; 0.026
L1	0.26 (0.03)*	0.16 (0.02)*	0.25 (0.03)*	0.17 (0.02)*	-1.99; 0.07	0.26	-1.81; 0.11	0.24	0.59; 0.54	0.08	2.08; 0.036	2.24; 0.025
m.n.	-0.04 (0.03)	-0.17 (0.09)	-0.05 (0.03)	-0.15 (0.09)	-0.28; 0.52	0.02	-0.10; 0.82	0.01	-0.83; 0.36	0.07	-0.47; 0.68	0.77; 0.012
un.imb.	33.90 (1.62)	41.66 (2.62)	33.91 (1.63)	41.16 (2.52)	0.13; 0.87	0.02	-0.06; 0.94	0.01	1.65; 0.09	0.22	-1.76; 0.043	1.14; 0.31
c.imb.	-14.61 (2.25)	-15.11 (3.36)	-6.77 (2.34)	-22.58 (3.12)	2.04; 0.018	0.28	-0.38; 0.63	0.05	-2.63; 0.007	0.36	0.32; 0.69	0.29; 0.81

m.n.= most negative electrode; **un.imb.**= uncompensated imbalances; **c.imb.**= compensated imbalances; **All**= all electrode recording sites (L5, L2, and L1 pooled); **<50 yrs**; **>50yrs**= participants' age groups (in years); **SE**= Standard error; **p**=P-value; **t**=t-statistic; **d**=Cohen's d; n= number of participants for whom a full set of electrode recordings was available;
* = significant change (p< 0.0025 = Bonferroni corrected significance level for the 5 levels and 4 subgroups = 0.05/20

Figure suppl. S1: Graphic illustration of the Monte Carlo simulation considering different effect sizes.

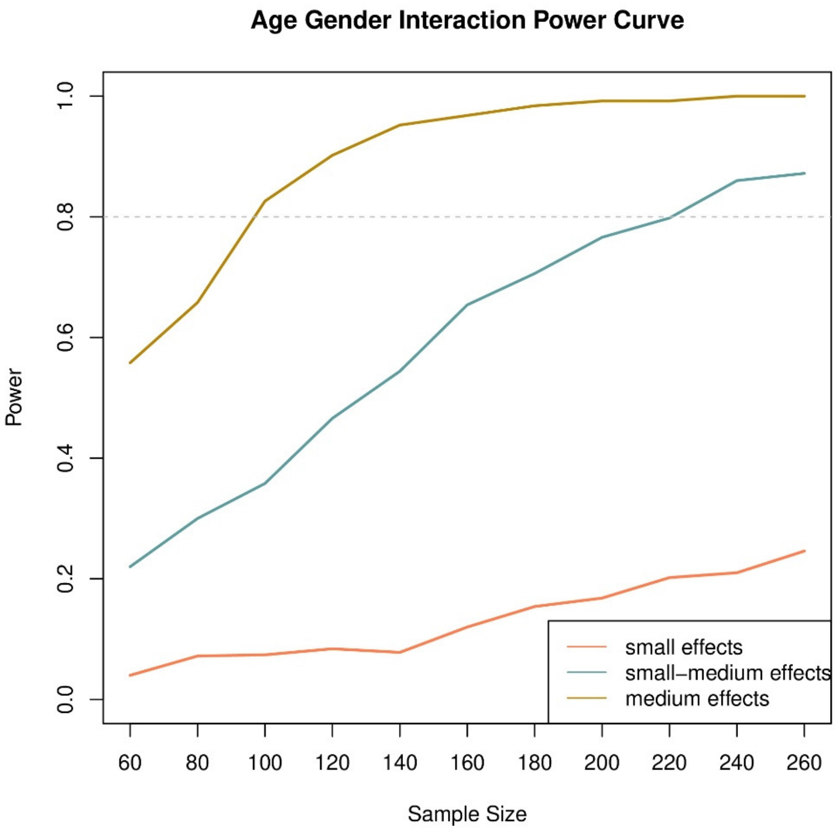

Figure suppl. S2: This figure shows the electrode recording site that revealed the most pronounced/ negative IMDF-SEMG fatigue slope normalized to the onset when the individual was retested on a second or third examination day. The arrows indicate how the electrode site depicting the most IMDF-SEMG fatigue changed between days.

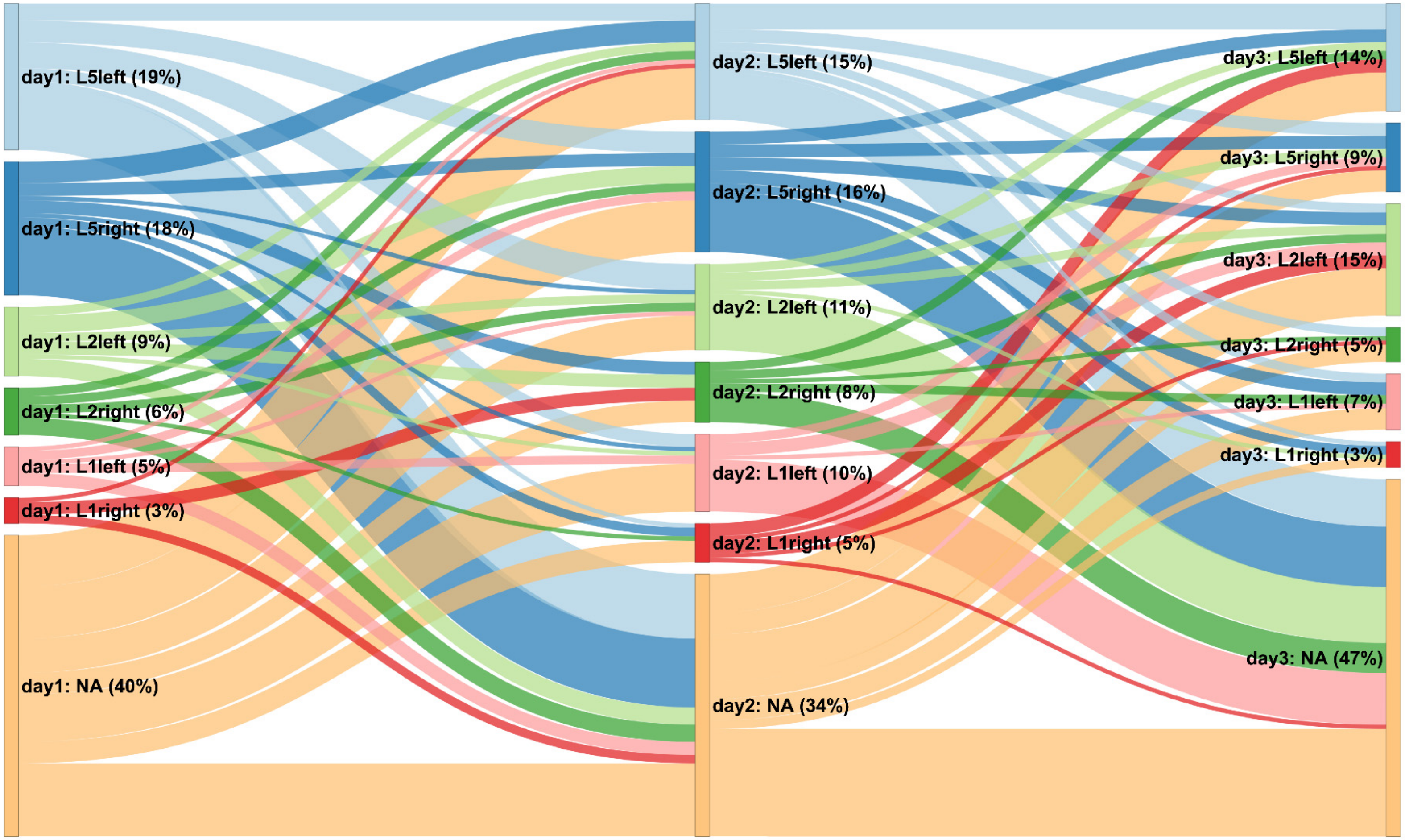

Supplement: Supplementary file 1 [file bioengineering-10-00300-s001.zip › bioengineering-2143878-supplementary.pdf]
